# Supplementary material for: Territorial differences in the spread of COVID-19 in European regions and US counties
Source: PLoS One. 2023 Feb 8;18(2):e0280780. doi: 10.1371/journal.pone.0280780 (PMC9907802; doi:10.1371/journal.pone.0280780)
Supplement: Attachment — Submitted filename: Replies to reviewers.docx [file pone.0280780.s002.docx]

| **Editor** |  |
| --- | --- |
| 1) In the review 2, you will find the request to put more attention (small discussion) about urban/rural definition (point 10 of his review), it is essential to refer to the EU+UN-Habitat+OECD work on urban/rural delineations on which I guess you based your typology? - Applying the Degree of Urbanisation — A methodological manual to define cities, towns and rural areas for international comparisons — 2021 edition, DOI: 10.2785/706535 A paragraph on this discussion would be appropriate as it is fundamental in your model. For a comparison of different approaches of "what is a city?", I would suggest a synthesis that I made recently (But I would not want to oblige you to quote my own work): - Rozenblat C. (2020). Extending the concept of city for the delineation of large urban regions (LUR) for the cities of the world, Cybergeo, [https://doi.org/10.4000/cybergeo.35411](https://priv-bx-myremote.tech.ec.europa.eu/v3/__https:/doi.org/10.4000/,DanaInfo=.ausngikku0nJn0z,SSL+cybergeo.35411__;!!DOxrgLBm!WOyZB7fjhrf0cd-UFFkE1veupc4el3emRfeNiUeB-MyjxXPm_fB71EkjdHnwg3sW7jDKJloH$) | In the data section, we have added the definition and some details on the urban/rural classification used in the analysis, as well as key references on the official Eurostat methodology. We have also cited Rozenblat C. (2020) for a more general overview of a city' s delineation. |
| 2) for the approach on mobility: I think that you should distinguish the "local mobility" that you consider, to the "global" connectedness that could be evaluated by the air passengers flows of airports. I think that urban areas are also previously affected because they are highly globally connected. For this discussion I woudl suggest: - Balcan, D., Colizza, V., Gonçalves, B., Hu, H., Ramasco, J. J., & Vespignani, A. (2009). Multiscale mobility networks and the spatial spreading of infectious diseases. Proceedings of the National Academy of Sciences, 106(51), 21484-21489. | We have added the suggested reference in the discussion of the results for Table 1 |
| 3) As a spreading infection, the discussion could interprete also (with some limits) the COVID diffusion processes from urban to semi-urban and rural areas. | We have added a sentence “*The fact that there was a lot of mobility …. cities to intermediate and rural areas*” in the Section “Mobility is higher in urban regions”. |

| **Review 1** | |
| --- | --- |
| - L46: are there other possible indicators to quantify epidemic spreading? More background on using the reproduction number could be given for readers not familiar with standards in epidemiology. | Expanded the description of the calculation of Rt |
| - L53: citations for both R packages are not provided; more details on the estimation procedure could also be given for more clarity - for example is there a time-window on which the reproduction number is computed? In this case is there some optimal window size, and which size is used here? | Added citations and expanded the description of the calculation of Rt |
| - A discussion on data quality and possible biases (for cases, mortality, and mobility) would be useful to add. | Biases of cases and mortality data are described in the text “This indicator (the excess mortality) has the downside of including fatalities not necessarily linked to COVID-19, such as those caused by the saturation of hospital capacity, but has the advantage of being less influenced by the underestimation of the real infection rate due to asymptomatic cases or differences in testing strategies over time and regions”  Biases of Mobility data. We have added a description of possible biases in the text “The limitation of our proposed indicators...” |
| - Linear statistical models are used here; could non-linear relationships be also considered? | In the revision we used the Log of the population and the population density variables, and the results have been significantly improved. |
| - The results comparing Europe and US contrast with the rest of the results focusing on Europe which are more detailed - furthermore urban contexts and definitions of cities are quite different in Europe and the US, so the comparison may not be that straightforward. I would suggest to put US counties as supplementary material, and focus the paper on Europe with robust and comparable results. | We agree that the comparison between US and EU is not straightforward but we also believe that what happened in US during the second wave where rural areas showed remarkably higher Rt values in respect of urban areas is an important and counterintuitive finding which would deserve prominence in the main text. |
| - L241: using instrumental variable to disentangle causalities between mobility and reproduction number is suggested; more details could be provided, in particular which kind of variable could be used? | The main objective of this analysis is to examine the rural-urban territorial differential in virus spread during the first and second waves of the pandemic. We are aware of the growing body of empirical studies examining the link between mobility and virus cases through the instrumental variables technique to untangle causality in the relationship (see for example Glaeser et al. (2020) and Kapoor et al. (2020) who use teleworkable and essential residential share travel and precipitation as instruments for mobility, respectively). An in-depth examination of the link between mobility and Rt through the instrumental variables technique is beyond the scope of this paper. However, to mitigate this potential problem, we use the mobility variables lagged by three weeks compared to the Rt values |
| - L244: as lagged regression are used, Granger causality tests could be provided here, or a study of lagged correlations. | Granger causality test requires a different setup, in particular a VAR specification of the model, which is not admissible because the timeseries involved are not stationary in the first place. So we performed a correlation analysis which is also summarized in Figure 6. |
| - Although mobility data can not be made publicly available for privacy reasons, source code should be provided (with synthetic mobility data for example) for replication on other case studies. | We made publicly available all data used for the Table1 and Table2 as well as the R code to produce the nine models presented in the respective tables. We added a random noise (non negative and skewed) to the mobility data since due to their commercial sensitivity we cannot published them. Clearly, the results of the analysis will not match with those in the paper, but all the code is reproducible. Interested researchers who wish to reproduce the regressions with mobility variables can use mobility data from other sources such as the Google mobility data. |

| **Review 2** | |
| --- | --- |
| 1) The authors should include a brief summary of the parameters used while calculating Rt. Because the authors aggregate to weekly averages, it is important for readers to understand that these are sliding windows. | In the methods section we added more details on the calculation of Rt and specified that this is indeed on a sliding window |
| 2) The authors should justify their choice of 9 regression models and provide statistics to compare between models. If drawing conclusions from multiple models, multiple comparison correction should be employed. | Although ours is not a truly multiple comparison setup, we agree with the referee that more than individual statistics of each coefficient should be reported. Indeed, we added also the F test and AIC statistic for each model. The evidence seems to be confirmed.  In the text we explain better now the rationale behind the choice of the models which is essentially to test the link between different Rt and degree of urbanisation. |
| 3) The authors include a model called “Internal pca” which is not described in the caption or mentioned in the main text or methods. | This is the mobility indicator divided by the population. We corrected the variable name to Internal per capita. |
| 4) The authors measure pandemic onset by the number o days between the beningng of 2020 and when the 20th confirmed case occurred. How was this number chosen? Are the results sensitive to different choices? | The choice of the threshold of 20 cases was determined by the need to condition results on the onset on sporadic events. We performed a sensitivity check using thresholds between 1 and 500 cases and the results showing a median earlier onset in urban regions in respect of intermediate and rural ones hold for all thresholds (Figure below).   |
| 5) When comparing differences in Rt, mobility, and excess mortality, across urban-rural gradients, the authors should employ appropriate statistical tests and multiple comparison corrections (they certainly have enough data). | We changed Figures 2, 3 and 4, respectively showing time series of Rt, mobility and excess mortality, with new ones including confidence intervals in addition to median values. The purpose of these figures is to provide a descriptive representation of rural-urban differentials before moving to a more formal assessment through regressions.  Although with this new visualisation the comparison across groups of regions by degree of urbanisation and over time is more cumbersome the non-overlapping confidence intervals provide preliminary clear indication of the presence of rural-urban differentials in certain periods of the pandemic. |
| 6) The methods section claims that the excess mortality time series spans from 2001 to 2015 but the results section claims that the baseline was calculated from data between 2011 and 2019. The authors should clarify and clearly state how the baseline was calculated (and with which data). | We corrected the text specifying that the time series start between 2001 and 2015, depending on the MS. For the model to calculate the baseline mortality we used all the time series for each country up to 2019. The reference to 2011 was a mistake. |
| 8) I would suggest the authors use log-population instead of population in their regression models as that is often a better indicator of ecological measures (e.g. Rt and mobility). | We used log both for the “Population” and the “Population density” variables. The R2 has been increased by doing so.  We also run a random forest model, which is a non-parametric and nonlinear model, and extracted the relative importance of the variables. The results are given below and confirm the importance of the mobility in the rural urban classification as we have seen in the regressions presented in Table 1 and Table 2.   |
| 9) In the introduction the authors cite three papers which look at the relationship between city population and the spread of covid-19, and claim that these studies refer to population density. In fact, these studies are all based on urban scaling theory (Bettencourt, 2013) and specifically aim to understand the role of social network density in covid-19 spread. The distinction between social network density and population density is an important theoretical one and should be acknowledged. | We have modified a section of the introduction to make the difference between studies analysing population density and those analysing the intensity of social contacts on virus spread clearer. |
| 10) Also in the introduction and the results it is crucial that the authors discuss the definitions of urban, intermediate, and rural regions being used. There are a number of different ways to decide what is a city (Taubenböck, 2012), and these choices might impact the interpretation of results. | The definition and classification we use to distinguish European regions and American counties according to urban-rural typology are now provided in the data section together with links and a few references. This is also mentioned in the introduction. There are several ways to decide what a city is, and we follow the official classification of European regions developed by Eurostat and implemented by the JRC. |
| 11) There is inconsistency in how the terminology for the three mobility indicators. I would suggest sticking to a single choice for each indicator to improve clarity. | We have harmonized the definition of the variables. |
| 12) The authors should justify the comparison of models with different temporal shifts and put forth a hypothesis for how the results of these comparisons might indicate causality. As it stands, their data does not suggest any causal relationship between reproductive numbers and mobility. Part of this is that the methodology needs to be more clear. | In the regression analyses, we use the mobility variables lagged by 3 weeks relative to the Rt value to account for the delay between the infection and the confirmation of the positive case. Figure 6 presents the regression coefficients obtained from the tests of alternative lag periods. This study is not aimed at a causality analysis between mobility and Rt, however we quantify the different time-lag effects to detect their potential influence on transmission, which is useful for COVID-19 monitoring policies. In a note, we pointed out that a specification linking Rt values to mobility may suffer from reverse causality. |
| 13) The authors say that “mobility changes may respond endogenously to Rt values”. I question whether people actually respond to Rt values rather than perceived risk. It might be the case the people are checking Rt values on the internet and changing their behavior, but if so citations or additional evidence are needed. | The sentence "mobility changes may respond endogenously to Rt values" has been removed from the text. An in-depth causality analysis of the link between mobility and Rt values is beyond the scope of our paper. |
| 14) Without proper statistics for model comparison, the discussion of the meaning of regression coefficients is difficult to justify. The authors should also address the fact that their regression models explain such a small proportion of the total variance in reproductive numbers. | We have improved the proportion of variance that is explained by using a new mobility indicator (mobility change compared to a reference date). We also used log of the “population” and “population density variables”.  In addition to R2 we also estimated AIC and F - Statistics. |
| 15)  Figure 2 should have some sort of indication of the error in the calculated Rt values.  Figure 3 is confusing and the excess mortality axis should be color to match the bars.  Figure 5 is missing a description of what the color bar means.  Figure 6 is missing an x axis label | We modified Figure 2 to include 95% confidence intervals in addition to median Rt values. We refer to the regression results for a more formal statistical assessment of the rural urban differences.  We modified Figure 3 by dividing it in two plots.  We have added a description of what the color bar means in Figure 5.  We added in Figure 6 the x axis label. |
| 16) The authors state that “mobility explains entirely the differences between the three groups of regions”. While it is true that differences in Rt by regional characterization are non-significant when conditioning on mobility, this statistical model treats regions with different levels of urbanization similarly. I suggest that the authors run an additional model where level of urbanization is treated as random effect to allow for different relationships between mobility and Rt in the different types of regions: a 50% reduction of mobility in a sparsely populated rural area may have only a very small effect of Rt, while a similar reduction in mobility might drastically impact spread in a large city. | we run a random effect model using the degree of urbanization as random effect to test the different relationships between mobility and Rt. The results, given in the below Figure seems to be confirmed in the SI.   |
