# Supplementary material for: Territorial differences in the spread of COVID-19 in European regions and US counties
Source: PLoS One. 2023 Feb 8;18(2):e0280780. doi: 10.1371/journal.pone.0280780 (PMC9907802; doi:10.1371/journal.pone.0280780)
Supplement: Attachment — Submitted filename: Reply to reviewers2.docx [file pone.0280780.s003.docx]

| **Reviewer 3** |  |
| --- | --- |
| In my opinion, this work should be accepted as is, as the authors revised the paper according to the previous reviewers’ comments and answered all the questions convincingly. Given that the produced results differ for different waves it would be interesting to observe the application of the same analysis to the 3rd or 4th wave. Moreover, another extension of this work would be to focus the study on the reasons of the differences between the waves. | The paper was originally submitted in March 2021 and has taken a long review process. During this long period the pandemic has progressed into 3rd or 4th waves.  We have checked the mortality data for the EU and our findings about the lack of rural-urban differences are confirmed in the subsequent periods.  As we indicate in the discussion major difference between first and second wave is linked to the widespread territorial diffusion of the infections following its initial onset in cities. We expect that similar effect continue to explain the rural-urban inversion in the later periods. We will consider the suggestions for further work. |
| **Reviewer 4** |  |
| 1. What is the motivation to compare the COVID-19 transmission in Europe and the US? What’s the definition of NUTS3 region? What is Rural-Urban Continuum Codes applied to the US? Although you indicated the detail in the footnote, I suggest giving brief description about this information. | We considered two areas of the world for which there was sufficiently detailed data on COVID19 cases at weekly and regional levels and with a harmonised classification allowing a comparison according to rural-urban typologies.  We add further details on the classification of regions and rural-urban typologies in the EU and US. |
| 2. What kind of regression you applied in the analysis?? The approach should be mentioned in the method but you described OLS and other detail in the result. | We moved the description of the regressions in the methods section. |
| Why do you include 3-week lags in the model?? Any scientific evidence to support the 3-week duration? | In the regression analyses, we use the mobility variables lagged by 3 weeks relative to the Rt value to account for the delay between the infection and the confirmation of the positive case.  A sensitivity analysis of the lag is given in Figure 6. We selected a lag of 3 weeks in the past which is maximising the positive coefficient of mobility during the second wave.  Positive lags produce as expected negative coefficients since mobility is reacting through restrictions rather than driving infection.  We add more details to describe the choice of the lag in the text.  Also, for Italy, [Carteni et al. (2020)](https://www.sciencedirect.com/science/article/pii/S0264999322003200#bib15) report that trips made three weeks earlier are the main determinants of new daily cases.  With data on counties in the US between January and April 2020, [Badr et al. (2020)](https://www.sciencedirect.com/science/article/pii/S0264999322003200#bib5) show that declining mobility is strongly correlated with lower COVID-19 case growth rates, and that the impact may not be evident for up to three weeks. This is consistent with the incubation period of the first virus variants plus the additional time for reporting. |
| 3. Although the title indicates the analysis will compare Europe and the US, it seems that the analysis about US only appeared in Figure 1 and 2. The excess mortality, mobility analysis only focused on Europe. The authors need to describe it clearly. | We added a sentence clearly stating that the part on mobility covers only the EU. |
| 4. Table 1 and 2. What is the number in the parentheses of each coefficient? P-value? | We add in the caption of the tables a sentence specifying that numbers in parentheses represent the standard errors. |
| 5. The adjusted R-squared values in the regression models are very low. Are you able to draw reliable conclusions from the models?? | Our purpose is not to predict the spread of the pandemic. For this we agree that there would be a need to achieve higher R-squared. The prediction of the spread of infections rather than simple OLS would require proper epidemiological models able to capture the typical temporal dynamics of epidemics. In our exercise we ignore these temporal dynamics and focus on the territorial differences and this explains the relatively low R-squared. The main aim of the paper is to see if the difference between rural and urban is significant and what is the role of mobility in explaining such difference. We tested alternative formulation of the model including as control variable a time dimension. This increasing dramatically the value of R-squared reflecting the time dependency in Rt values but has the downsize of cancelling and adsorbing most of the effect from our variables of interest. |
